# Biodegradable mesoporous manganese carbonate nanocomposites for LED light-driven cancer therapy via enhancing photodynamic therapy and attenuating survivin expression

**DOI:** 10.1186/s12951-021-01057-2

**Published:** 2021-10-09

**Authors:** Lihua Li, Lingling Chen, Ling Huang, Xiangling Ye, Zefeng Lin, Xiaoming Wei, Xianfeng Yang, Zhongmin Yang

**Affiliations:** 1 The State Key Laboratory of Luminescent Materials and Devices; Guangdong Provincial Key Laboratory of Fiber Laser Materials and Applied Techniques, Analytical and Testing Center, South China University of Technology, Guangzhou, 510640 Guangdong China; 2 Guangdong Key Lab of Orthopedic Technology and Implant, General Hospital of Southern Theater Command of PLA, The First School of Clinical Medicine, Southern Medical University, Guangzhou, 510515 China; 3 The Fifth Clinical Medical College, Guangzhou University of Chinese Medicine, Guangzhou, 510095 China

**Keywords:** Triple negative breast cancer, Mesoporous MnCO_3_ nanocubes, LED light responsive, O_2_ and CO_2_ release, Reactive oxygen species

## Abstract

Triple-negative breast cancer (TNBC) is one of the most daunting diseases, low toxicity and efficient approaches are in urgent demand. Herein, we developed degradable mesoporous manganese carbonate nanocubes (MnCO_3_ NCs), incorporated with survivin shRNA-expressing plasmid DNA (iSur-pDNA) and riboflavin (Rf), namely MRp NCs, for synergistic TNBC therapy. The MnCO_3_, itself, could generate O_2_ and CO_2_ under H_2_O_2_ and thus relieve the hypoxia and acidic tumor microenvironment (TME). Furthermore, the MnCO_3_ NCs exhibited high Rf loading capacity and iSur-pDNA delivery ability after polyethyleneimine modification. Specifically, MRp NCs decompose in TME, meanwhile they deprived the endogenous expression of survivin gene and significantly amplified the generation of reactive oxygen species after exposure to LED light, resulting in serious tumor destruction. The multifunctional MRp NCs with LED light-driven characters are able to provide a high efficiency, low toxicity and promising strategy for TNBC therapy.

**Supplementary Information:**

The online version contains supplementary material available at 10.1186/s12951-021-01057-2.

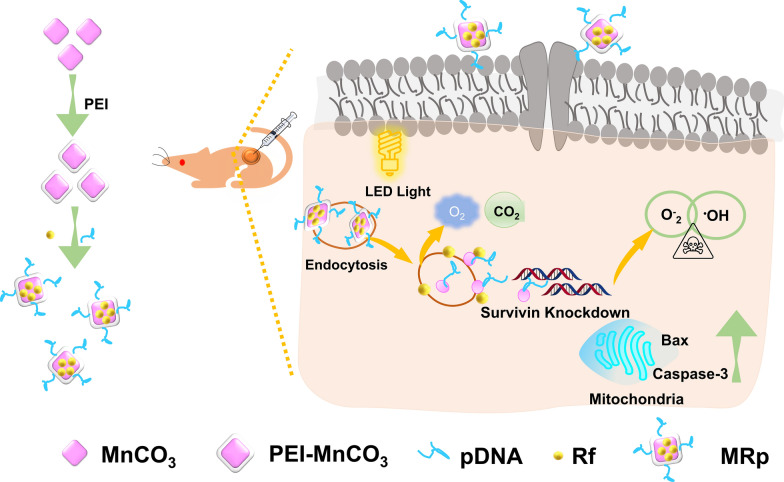

## Introduction

Triple-negative breast cancer (TNBC) is an important and intractable subtype of breast cancer due to the lack of biomarkers and its high metastasis [1]. There was no significant progress in TNBC treatment during the past decades [2]. Traditional chemotherapies are still the main approaches for TNBC therapy, but they exhibit high toxicity and low efficiency, resulting in poor life quality and low survival rates. It is urgent to develop new approaches with high safety, low toxicity, and high efficiency to deal with TNBC [3].

Photodynamic therapy (PDT) [4] and chemodynamic therapy (CDT) based on reactive oxygen species (ROS) provide new alternative opportunities for cancer therapy [5]. They exhibit high selectivity in cancer theranostic [6] and could be activated by inner (e.g., low pH, abundant glutathione, and H_2_O_2_) [7, 8] or external stimulus (light, magnetic field, and heat) [9–11] compared with chemotherapy. Manganese-based nanoparticles have been widely reported for cancer theranostic because of their excellent tumor microenvironment (TME) responsive characters and potential CDT effect [12]. These features selectively damage the tumor cells while protecting the normal cells because they are restrained in the specific tumor regions. We previously found Bi@MnO_x_ nanoparticles could respond to both inner and external stimuli, exhibiting a mutual reinforcement for cancer therapy [13]. Recently, researchers have focused on the catalytic reaction of MnOx [14], and various ROS-based nanozymes have been developed for cancer therapy (e.g., MnOx-SPNs [15], Au-MnO [16], and MnOx [17]). However, it is still difficult to tackle with TNBC only using CDT/PDT approaches.

**Scheme 1:**
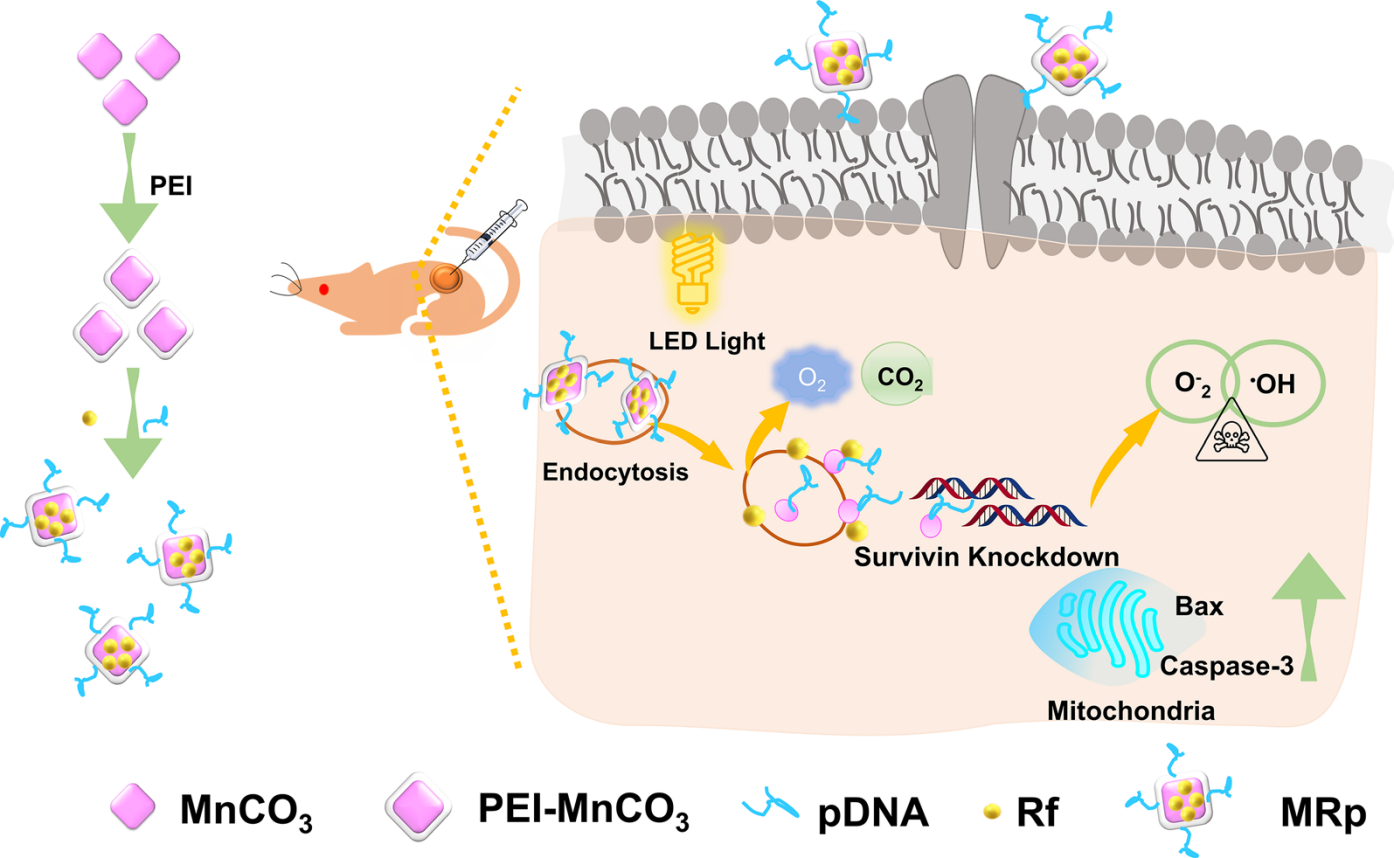
Schematic illustration of MRp NCs for synergistic TNBC therapy

To address these issues, we developed MnCO_3_/Rf/pDNA nanocomposites (denoted as MRp NCs) which consisted of mesoporous MnCO_3_ nanocubes (NCs) loading with riboflavin (Rf) and survivin shRNA expressing plasmid (iSur-pDNA) for combined TNBC therapy. Rf, as a necessary nutrient for the human body, could also work as a photosensitizer. Its ROS production was significantly amplified in the presence of MnCO_3_ NCs. Moreover, the polyethyleneimine (PEI) modified MnCO_3_ NCs could efficiently deliver iSur-pDNA to 4T1 cells for survivin gene silencing. The MRp NCs illustrate multiple roles in TNBC therapy: (i) as TME ameliorative agents for improving tumor acidity and hypoxia; (ii) as a biodegradable drug and pDNA carrier; The high surface potential enables the PEI-MnCO_3_ NCs with high pDNA transfection efficiency; (iii) as an assistant for combined TNBC therapy. The MRp NCs can be decomposed under simulated TME solution, resulting in the release of Mn^2+^, O_2_ and CO_2_ for enhancing PDT and CDT, moreover, the generated O_2_ and CO_2_ could also destroy the tumor tissue, and the delivered pDNA could deprive the survivin gene, thus enhancing tumor cell destruction (Scheme 1).

## Experiment section

### Materials

Cetyltrimethylammonium bromide (CTAB), MnCl_2_·4H_2_O, 1-butanol, cyclohexane, KHCO_3_, NH_4_HCO_3_, polyethyleneimine (PEI, 10,000 KDa), and ethanol were purchased from Aladdin Co., 1,3-diphenylisobenzofuran (DPBF), Ltd (Shanghai, China). Riboflavin, 30% hydrogen peroxide (H_2_O_2_), Calcein-AM, propidium iodide (PI), and cell counting Kit-8 (CCK-8) were purchased from Sigma-Aldrich (USA) Phosphate buffer saline (PBS), fetal bovine serum (FBS), penicillin/streptomycin (PS) and Dulbecco’s modified Eagle’s medium (DMEM) were purchased from Gibco Life Technology (AG, Switzerland). 4% Paraformaldehyde fix solution and GSH/GSSG assay kit were obtained from Beyotime. Hypoxia detection kit was bought from Enzo Biochem. Inc. (USA). L-buthionine sulfoximine (L-BSO) was obtained from Meilun Biotechnology Co., Ltd (Dalian, China). All of chemical reagents were used as received without further purification. The *Escherichia coli* containing iSurvivin pDNA purchased from GenePharma Co., Ltd. (Shanghai, China). The iSur-pDNA vector were amplified in *Escherichia coli* and isolated with an EndoFree Plasmid Mega Kit (Tiangen Biotech Co., Ltd., Beijing, China). The forward primer and reverse primer sequences of survivin were: Sur-sense: 5’-AATCATGAATCCATGGCAGCCAG-3’ and the reverse primer 5’-AAGAATTCATGGGTGCCCCGA-3′ [18]. β-actin sense: 5′-CCA ACC GCG AGA AGA TGA-3′ and the reverse primer 5′-CCA GAG GCG TAC AGG GAT AG-3′, respectively.

### Preparation of MnCO_3_ NCs

The MnCO_3_ synthetic process was according to our previous work [19]. CTAB (2 g), MnCl_2_·4H_2_O (10 mmol) were mixed in 2.0 mL water, 3.0 mL 1-butanol and 60 mL cyclohexane, and then the mixture was vigorously stirred at room temperature, named as A solution. CTAB (8 g), of KHCO_3_ (19 mmol), NH_4_HCO_3_ (1 mmol), 8.0 mL water, 3.0 mL 1-butanol and 240 mL cyclohexane were mixed and vigorously stirred in container B. After magnetic stirring for 1 h, solution A was added to container B under continuous stirring. After reacted for another 0.5 h, the solution was centrifuged at 8000 rpm for 10 min to remove the supernatant. The final MnCO_3_ was washed with ethanol and dd H_2_O three times, and then the precipitates were extracted several times using methanol with 1% NaCl to remove the redundant CTAB.

### Modification of MnCO_3_ NCs

Surface modification of MnCO_3_ NCs with amine-containing PEI was followed below, 0.1 g MnCO_3_ NCs were dispersed in 100 mL ddH_2_O with vigorously stirring, then 0.1 g PEI was added to the solution. The mixture was stirred at room temperature for another 2 h. The PEI-MnCO_3_ NCs were collected by centrifugation (10,000 rpm, 10 min) and washed with water 3 times to remove the redundant PEI.

### Characterization

The powder X-ray diffraction (XRD) patterns were collected with a Siemens Kristalloflex 810 D-500X-ray diffractometer using Cu Kα irradiation (λ = 1.5406 Å). High-resolution transmission electron microscopy was taken on a field emission scanning electron microscope (JEOL JEM-2100F, Japan). Zeta potential and hydrophilic size were measured using a zetasizer (Zetasizer Nano ZS, Malvern, UK). UV–vis-NIR absorption spectra and absorbance were examined using a multifunctional microplate reader (TECAN, infinite M200 PRO, Swiss).

### Drug loading

PEI-MnCO_3_ NCs (500 μg) were suspended in 5 mL PBS solution, and then Rf was dispersed in the solution at a concentration of 100 μg mL^−1^. After stirring at 4 °C for 24 h, the solution was centrifuged. And the supernatant and precipitations were collected respectively. PEI-MnCO_3_/Rf NCs was named as MRf. The drug loading efficiency was calculated as below (Eq. 1):

1 $${\text{Drug loading efficiency }}\left( \% \right) = \frac{Rf(Total) - Rf(Superna\tan t)}{{Rf({\text{T}}otal)}}$$

### Extracellular O_2_ measurement

The O_2_ production from PEI-MnCO_3_ NCs in H_2_O_2_ solution was monitored by a portable dissolved oxygen meter (HANNA HI 2400). Briefly, different concentrations of PEI-MnCO_3_ NCs (0, 100, 200 μg mL^−1^) was added to 10 mM H_2_O_2_ PBS solution. The data was recorded every 5 s for 10 min using the portable dissolved oxygen meter.

ROS detection. ROS generation was detected by ESR. Typically, Rf, PEI-MnCO_3_ and MRf NCs (100 μL, 0.5 mg mL^−1^) were mixed with H_2_O_2_ (100 μL, 16 mM) containing the trapping agent 5, 5-dimethyl-1-pyrroline-N-oxide (DMPO, 10 μL, 10 mM). Then, the X-band ESR spectra were acquired by Bruker ELEXSYS-II spectrometer at 37 ℃. The raw MnCO_3_ and H_2_O_2_ were set as control.

### Extracellular ROS detection

1 mM fluorescent dye 2′,7′-dichlorodihydrofluorescein diacetate (DCFH-DA, Sigma, USA) was hydrolyzed to DCFH using NaOH (1 mM) for intracellular ROS detection. Rf (10 μg mL^−1^), PEI-MnCO_3_, and MRf NCs (100 μg mL^−1^**)** was added to 2 mM H_2_O_2_, DCFH (1 μM) was added to the above solution and the mixture was exposure to LED light for 10 min, then their emission was monitored using a microplate reader (Ex/Em = 488/525 nm).

### Biodegradation of MnCO_3_ in TME simulation solution

PEI-MnCO_3_ NCs were incubated in a solution of PBS (pH = 6.5) containing 2 mM H_2_O_2_ for 3, 12 and 24 h, respectively. The morphologic changes of the PEI-MnCO_3_ NCs were observed using TEM. In addition, PEI-MnCO_3_ NCs were incubated in PBS (pH = 6.5) for 10, 30, 60 and 120 min, the CO_2_ contents were assessed by meteorological chromatograph. After 2 h, the solution was centrifuged, the precipitates were analyzed using XPS.

### ^1^O_2_ measurement

^1^O_2_ generation was measured using a 1,3-diphenylisobenzofuran (DPBF) probe. PEI-MnCO_3_ NCs were incubated in a simulated TME solution **(**PBS (pH = 6.5) containing 2 mM H_2_O_2_). 2 μL of DPBF solution (10 mM, DMSO) was added to 200 μL above solution. The absorbance of DPBF at 410 nm was recorded every 2 min by a microplate reader.

### Cell lines

The mouse TNBC cell line 4T1 and L929 cells were obtained from American Type Culture Collection. 4T1-Luc cells were maintained in RPMI 1640 medium (Sigma) with 10% FBS and penicillin (100 U/mL) and streptomycin (100 μg/mL) (Invitrogen). L929 cells were maintained in Dulbecco’s modified Eagle’s medium (Sigma) with 10% fetal bovine serum (FBS, Gibco) and penicillin (100 U/mL) and streptomycin (100 μg/mL) (Invitrogen). The cells were cultured at 37 °C under a humidified atmosphere of 95% air and 5% CO_2_ and the medium was changed every 2 days.

### Cell viability

4T1 and L929 cells were seeded in 96-well plates with a density of 5 × 10^4^ cells per well, respectively. After culturing for 24 h, gradient concentrations of PEI-MnCO_3_, Rf and MRf (500, 250, 125, 62.5, 31.25, 15.6, 7.8, 0 μg/mL) were co-cultured with the cells for another 24 h. Then, MTT assay was measured according to the standard protocol.

### Transfection of pDNA

4T1 cells were seeded in 6-well plates with a density of 5 × 10^4^ cells per well. The medium was removed with fresh 1640 medium without FBS. All NCs were prepared by MRf/pDNA with a weight ratio of 15/1. Then 15 μL MnCO_3_/pDNA mixture was added to the 6-well plate co-cultured for 6 h. The medium was changed with fresh 1640 containing 10% FBS and 1% PS.

### Live/dead staining

4T1 cells were seeded on 24-well plates at a concentration of 5 × 10^4^ cells/cm^2^ under 37 °C with 5% CO_2_ for 24 h. 200 μL of PEI-MnCO_3_ (50 μg mL^−1^), Rf (50 μg mL^−1^), MRf (50 μg mL^−1^), MRp (50 μg mL^−1^) were added to the plate, then those groups were exposure to LED light or in dark for 10 min, respectively. After co-cultured for 24 h, the cells were subject to Live/Dead staining following the manufacturer's protocol (Sigma, USA) and imaged under a fluorescence microscope (DMI4000, Leica).

### Intracellular ROS detection

Intracellular ROS production was detected by DCFH-DA. In brief, 4T1 cells were seeded in 24-well plate (1 × 10^5^ cells per mL) and cultured overnight. Then cells were treated similarly as above (as live/dead staining). Finally, the cells were incubated with DCFH-DA probe (1 μM) for 15 min, washed with PBS and observed by fluorescence microscopy. Moreover, their quantitative analysis was using a multifunctional microplate reader (Ex/Em: 488/525 nm).

### Intracellular pH detection

The changes of intracellular pH were using an intracellular pH fluorescence probe (BCECF AM). Briefly, the 4T1 cells were treated with PEI-MnCO_3_ NCs (50 μg mL^−1^) as experiment group and medium as control group, then the cells were cultured with BCECF AM (5 μM) for 20 min, and their images were observed under fluorescence microscopy. And their quantitative analysis was using a multifunctional microplate reader (Ex/Em: 488/535 nm).

### Animals

Balb/c nude mice (6-week-old, female) were purchased from Guangdong Medical Lab. Animal Center. The protocol was approved by the Institutional Animal Care and Use Committee of General Hospital of Southern Theater Command of PLA.

### In vivo tumor therapy

5 × 10^6^ 4T1 cells were injected to the second breast nodule of the nude mice. After the tumors grew to a size of 50‒70 mm^2^, the mice were divided into 5 random groups (n = 4) undergoing different treatments: (1) PBS; (2) PEI-MnCO_3_; (3) Rf + LED light; (4) MR + Light; (5) MRp + LED light; The NCs were injected intratumorally into the 4T1-bearing mouse. The size of the tumors was measured every other day for 2 weeks. The tumor volumes were carefully measured every other day for 14 days by a caliper and calculated as Eq. 2

2 $$V = \frac{{{\text{ab}}^{2} }}{2}$$

where *V* (mm^3^) is the volume of the tumor, and *a* (mm) and *b* (mm) is length of tumor and width of tumor, respectively. Then the tumors were histologically analyzed by hematoxylin and eosin (H&E) staining.

## Results and discussion

### Characterization of MRp NCs

**Fig. 1:**
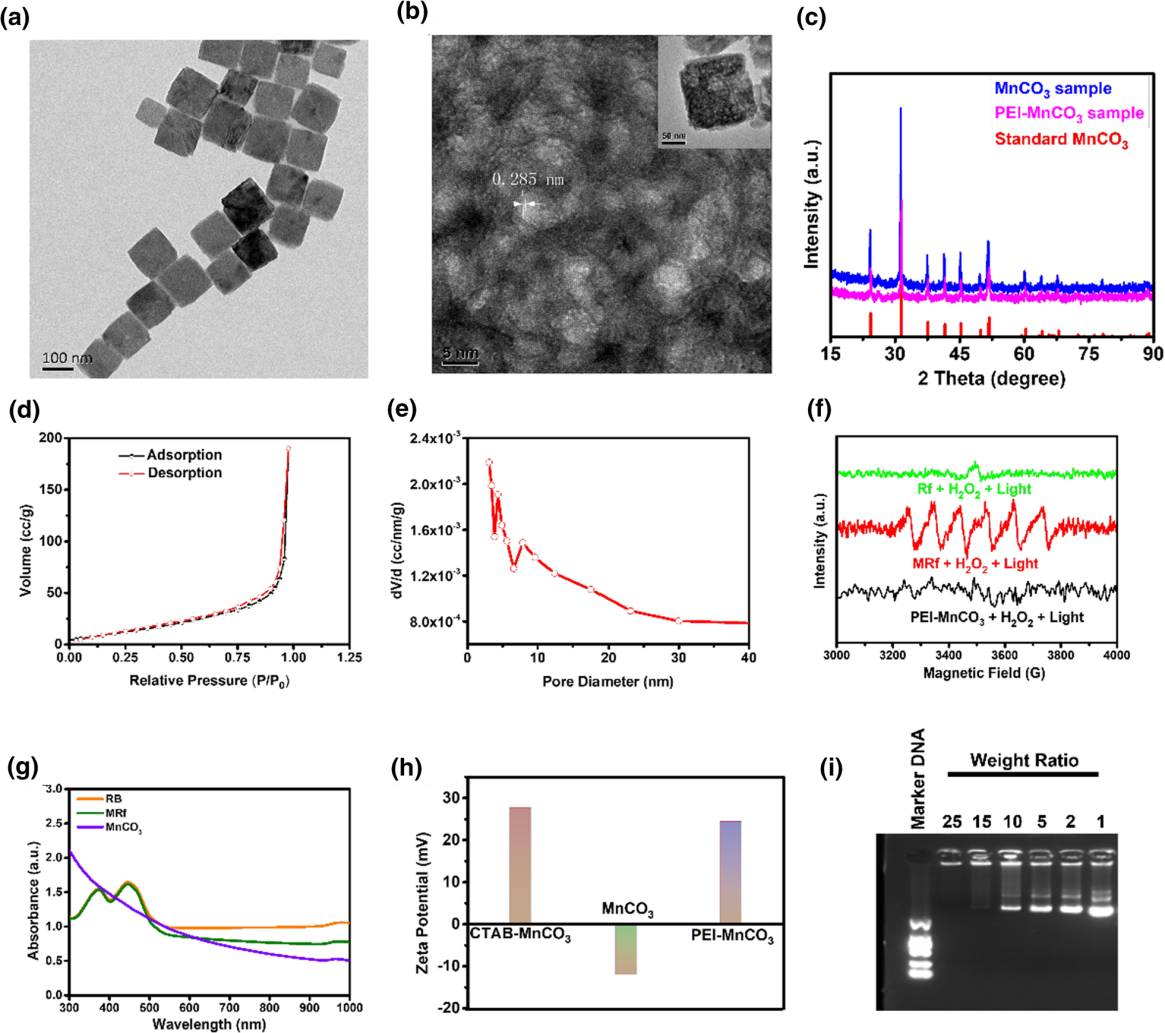
Characterization of MRp NCs. **a** Low magnification and **b** high resolution TEM images of MnCO_3_ NCs. Inset, TEM of the corresponding MnCO_3_ NC. **c** XRD spectra of MnCO_3_ NCs and PEI-MnCO_3_ NCs. **d** N_2_ adsorption–desorption isotherms and **e** desorption pore size distribution of PEI-MnCO_3_ NCs. **f** Zeta potential of CTAB-MnCO_3_, fine MnCO_3_ (washed out CTAB) and PEI-MnCO_3_. **g** Absorbance spectra of PEI-MnCO_3_, Rf and MRf. The MRf was almost overlapped with Rf, the loading efficiency was calculated to be 90%. **h** Agarose-gel electrophoresis of MRf and pDNA at different weight ratios. **i** Electron spin trapping (ESR) spectra of PEI-MnCO_3_, Rf and MRf with H_2_O_2_ (10 mM) under LED light using DMPO

Firstly, the monodisperse MnCO_3_ NCs were prepared by a microemulsion method according to our previous method [19]. Transmission electron microscope (TEM) image (Fig. 1a) revealed that the MnCO_3_ NCs had cubic-like morphology with the particle size of ca. 120 nm. As shown in Fig. 1b, high resolution TEM image with a typical individual nanocube inset revealed its highly porous nature and the marked lattice spacings of 0.285 nm which could be indexed to the (104) planes of MnCO_3_. X-ray diffraction (XRD) pattern was employed to detect the crystalline phase and purity of the samples. The results (Fig. 1c) revealed that the samples were pure rhombohedral MnCO_3_ (JCPDS Card No. 44-1472). In addition, the porous structure of the MnCO_3_ NCs was investigated by Brunauer–Emmett–Teller (BET) analysis. As depicted in Fig. 1d and e, the PEI-MnCO_3_ NCs exhibited high Brunauer–Emmett–Teller surface area (49.97 m^2^ g^−1^) and pore volume (0.293 cm^3^ g^−1^), respectively. The average pore size is about 3.41 nm according to the N_2_ adsorption–desorption isotherms. Zeta potential of the CTAB-MnCO_3_, fine MnCO_3_ (removal of CTAB) and PEI-MnCO_3_ were shown in Fig. 1f, illustrating the successful modification of PEI. The porous structure of the PEI-MnCO_3_ NCs endows them with excellent Rf loading capacity. As can be seen in Fig. 1g, the loading efficiency (w.t%) of Rf in PEI-MnCO_3_ NCs was calculated as high as 90%, confirmed by the absorption spectra. More importantly, the PEI-MnCO_3_/Rf (MRf) NCs presented a high binding ability to pDNA because of their high zeta potential. The binding ability was investigated by gel retardation assays (as shown in Fig. 1h), the results illustrated that the iSur-pDNA could be completely loaded onto MRf NCs at the weight ratios of 1:15. The mean hydrodynamic diameter of PEI-MnCO3 and MRp was 105–190 nm, 105–220 nm, respectively, determining by dynamic light scattering (DLS) measurement (Additional file 1: Fig. S1, ESI†). The changes indicated the successfully loading of Rf and pDNA. Moreover, we have assessed the ROS production ability of PEI-MnCO_3_. Rf and MRf under the same condition using 5, 5-dimethyl-1-pyrroline-N-oxide (DMPO) as trapping agent. Inspiringly, MRf NCs exhibited significantly enhanced ROS production than PEI-MnCO_3_ and Rf, respectively (Fig. 1i).

### TME responsive characters

**Fig. 2:**
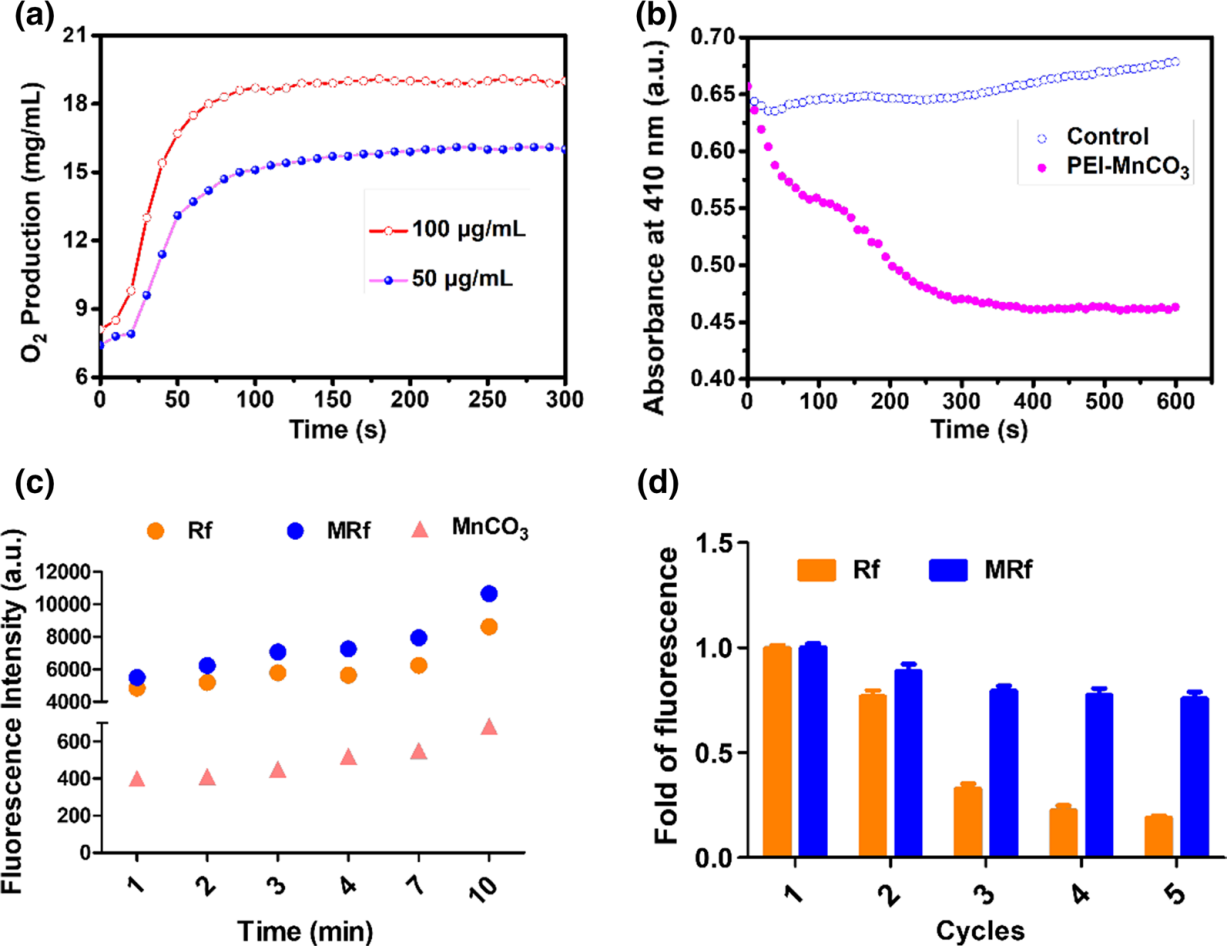
Enhanced CDT effect of MRf NCs. **a** O_2_ generation in different concentrations of PEI-MnCO_3_ NCs, the O_2_ contents were detected using a portable dissolved oxygen meter. **b** Dynamic changes of DPBF with PEI-MnCO_3_ NCs. **c** Time-dependent generation of ROS under LED light. **d** Stability of Rf and MRf under 5 cycles of LED light irradiation, data are represented as mean ± standard deviation (SD). All the above experiments were conducted in simulated TME (2 mM H_2_O_2,_ pH = 6.5, 2 mM GSH) solution

To verify the TME responsive characters of the PEI-MnCO_3_ NCs, we systematically analyzed their degradation characters, catalytic ability, and gas production under simulated TME (pH = 6.5, 2 mM H_2_O_2_, 8 mM glutathione). As can be seen in Fig. 2a, PEI-MnCO_3_ NCs worked as a H_2_O_2_ catalyst, which could catalyze H_2_O_2_ to produce O_2_ (Fig. 2a). Meanwhile, they decomposed slowly (Additional file 1: Fig. S2) in TME and produced CO_2_ (Additional file 1: Fig. S3) simultaneously. Their morphology changes were observed by TEM, illustrating a dynamic change of morphology, i.e., cubic-round-circle-dots, and PEI-MnCO_3_ NCs finally could be degraded into tiny round nanodots (Additional file 1: Fig. S2). Notably, the O_2_ production soared to 18.1 μg mL^−1^ while the CO_2_ production was in a relatively low speed (Fig. 2a, Additional file 1: Fig. S3). By contrast, there was negligible O_2_ production in commercial MnCO_3_ under the same condition (Additional file 1: Fig. S4). During the catalytic process, PEI-MnCO_3_ NCs degraded into small pieces (Additional file 1: Fig. S2), release Mn^2+^ and OH^−^ (Eqs. 3, 4), the OH^−^ ion is beneficial to improve the acidic TME while Mn^2+^ facilitate the Fenton reaction [20] (Eqs. 4, 5) in tumors. To prove this process, we carried out XRD to evaluate the NCs in simulated TME, the results indicated the partially degraded NCs were still MnCO_3_ without any impurities (Additional file 1: Fig. S5). The multivalence Mn in XPS spectra further confirmed the release of OH^−^ and the redox reaction in this process (Additional file 1: Fig. S6). As expected, we discovered ^1^O_2_ generation in PEI-MnCO_3_ NCs during degradation, which is beneficial for CDT (Fig. 2b and c, Eqs. 4, 5). Considering that Rf-mediated PDT consumes O_2_ in hypoxia TME, O_2_ produced by PEI-MnCO_3_ NCs may improve the efficacy of PDT (Eq. 6, Fig. 2b). Subsequently, we investigated whether the MRf produced more ROS compared with single Rf group under simulated TME solution when illuminated by white-light LED light. Both MRf and Rf generated ROS (Fig. 2c). Notably, the MRf could enhance the ROS production during the observing time (10 min) and this phenomenon could be repeated 5 times, indicating that PEI-MnCO_3_ NCs as drug loading carriers could significantly increase ROS production as well as protect Rf from photobleaching and photodamage [21] (Fig. 2d).

3 $${\text{MnCO}}_{{3}} {\text{ + H}}^{ + } {\text{ + H}}_{{2}} {\text{O}}_{{2}} \to {\text{Mn}}^{2 + } {\text{ + H}}_{{2}} {\text{O}}_{{}} {\text{ + CO}}_{{2}} \uparrow {\text{ + O}}_{{2}} \uparrow { }$$

4 $${\text{Mn}}^{2 + } {\text{ + H}}_{{2}} {\text{O}}_{{2}} \to {\text{Mn}}^{3 + } { + } \cdot {\text{OH + OH}}^{ - }$$

5 $${\text{Mn}}^{3 + } { } + {\text{ H}}_{{2}} {\text{O}}_{{2}} \to {\text{Mn}}^{2 + } + {\text{ O}}_{{2}} \uparrow + {\text{H}}^{ + } { }$$

6 $${\text{Rf}} {\text{ + O}}_{{2}} \mathop {\longrightarrow}\limits^{{{\text{Light}}}} {\text{Rf}}'\,^{{1}} {\text{O}}_{2}$$

Note: Rf’ is the excited Rf.

### Cellular ROS production and pH-responsive ability

**Fig. 3:**
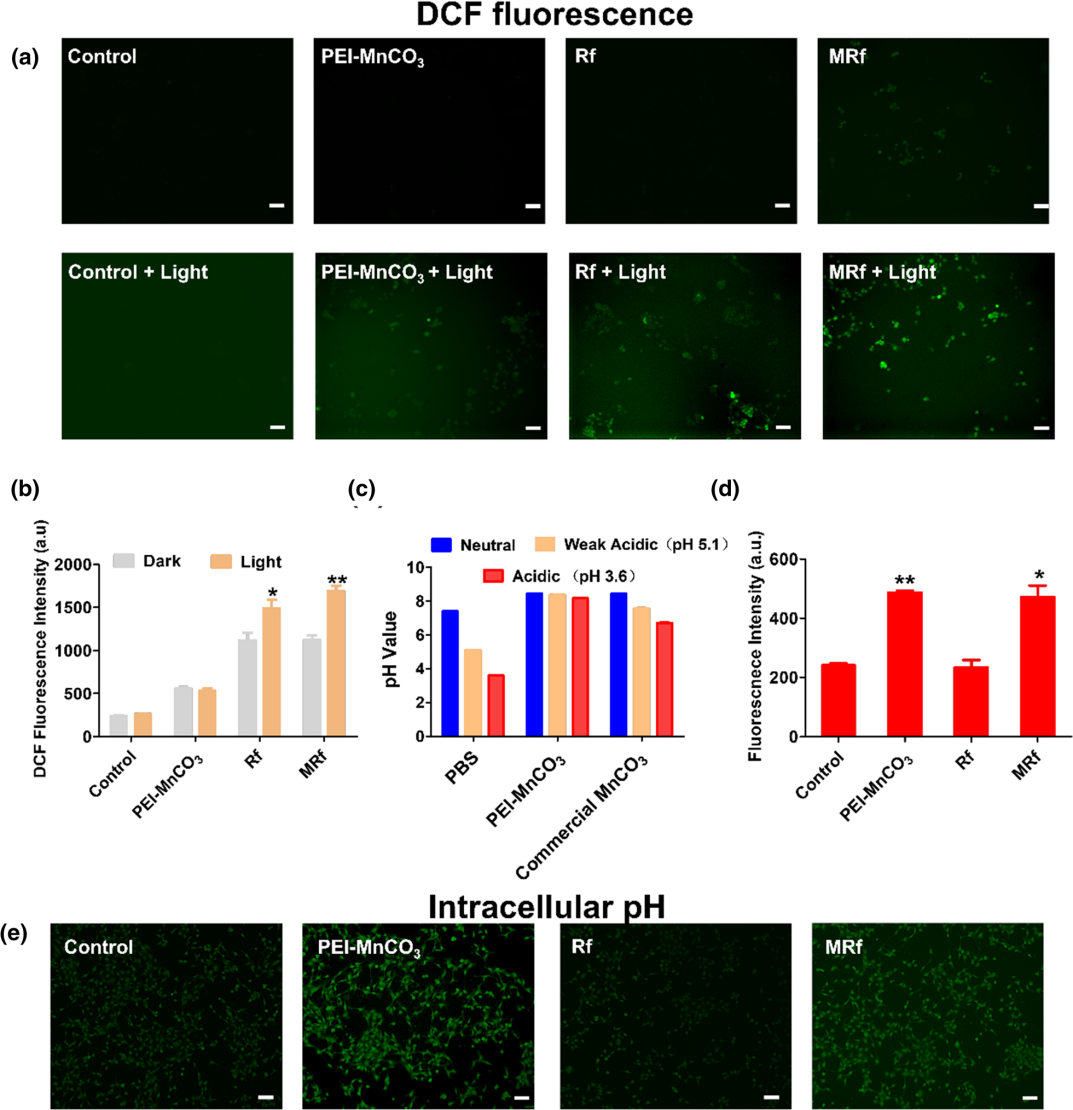
Tumor microenvironment responsive ability of MRf NCs. **a** DCF fluorescence (ROS level) images of 4T1 cells after different treatment. **b** DCF fluorescence intensity in different treatment groups (Ex/Em = 488 nm/525 nm). **c** pH changes of different pH PBS solutions after PEI-MnCO_3_ and commercial MnCO_3_ treatment. **d** BCECF fluorescence intensity in different treatment groups (Ex/Em = 430 nm/530 nm). Data are represented as mean ± SD; n = 4; Statistical significance was analyzed by the two-tailed Student’s *t*-test. *p < 0.05, **p < 0.01. All light treated groups were compared to the dark treated group in c. And all groups are compared to control group in e. **e** BCECF fluorescence (pH changes) images of 4T1 cells after different treatment

Thanks to the excellent performance of MnCO_3_-based NCs in TME, we then investigated their cancer-killing efficiency. Firstly, the intracellular ROS production was monitored by using the green probe, 2',7'-dichlorofluorescein diacetate (DCFH-DA). As shown in Fig. 3a and b, the cells treated with MRf + LED exhibited much stronger fluorescent intensity than Rf + LED and PEI-MnCO_3_ + LED groups. By contrast, the groups (control group, PEI-MnCO_3_, Rf and MRf) without LED illumination showed weak fluorescence. The results suggested that the ROS production capability of Rf could be significantly improved by PEI-MnCO_3_in vitro. Next, we tested the changes of intracellular acidity because of the OH^−^ release and pH-sensitive characters of PEI-MnCO_3_ NCs. Noteworthy, the synthesized MnCO_3_ NCs exhibited better pH stability than commercial MnCO_3_ (Fig. 3c), which may attribute to their high surface area and porous structure. The green pH probe (2',7'-bis(carboxyethyl)-5(6)-carboxyfluorescein, BCECF) was employed to investigate the intracellular pH changes of 4T1 cells after different treatments. The PEI-MnCO_3_ treated cells exhibited much stronger fluorescence intensity than the control group, suggesting they could improve the acidic environment in tumor cells (Fig. 3 d, e). Such an interesting pH improvement will help to destroy lysosomes and ameliorate TME ability, thus accelerating the death of cancer cells.

### The killing effect of MRp NCs on TNBC cells in vitro

**Fig. 4:**
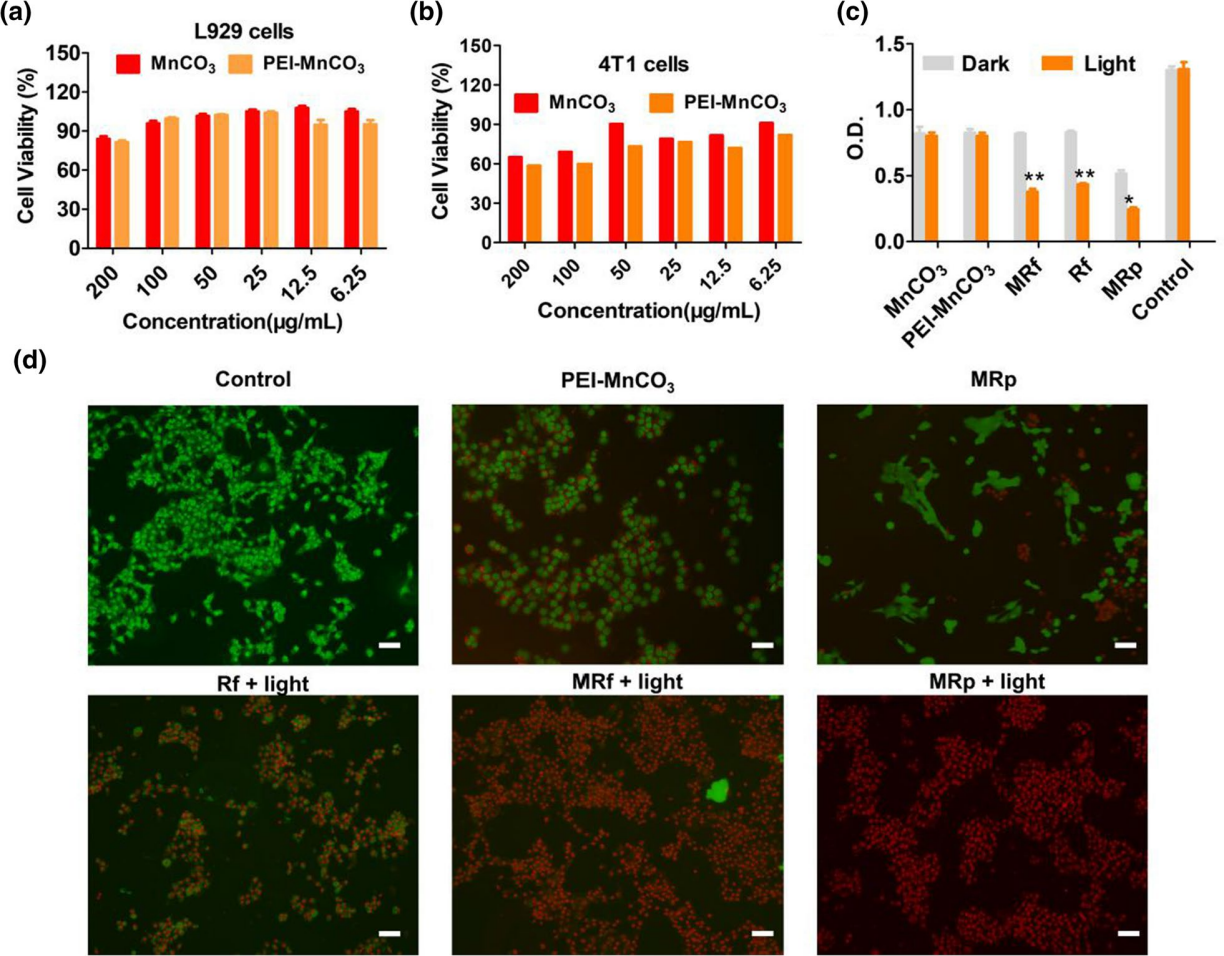
Cell viability. **a** Cell viability of L929 cells after treated with MnCO_3_ and PEI-MnCO_3_ NCs. **b** Cell viability of 4T1 cells after treated with MnCO_3_ and PEI-MnCO_3_ NCs. **c** Cell killing effect of different treatment on 4T1 cells in dark or under LED light. Data are represented as mean ± standard deviation (SD); n = 4; Statistical significance was analyzed by the two-tailed Student’s *t*-test. *p < 0.05, **p < 0.01. All light treated groups were compared to the dark treated group. **d** Live/dead staining of 4T1 cells with different treatment, scale bar = 50 μm

To verify the biocompatibility and anticancer effect of the synthesized NCs, MnCO_3_ and PEI-MnCO_3_ NCs with different concentrations were co-cultured with 4T1 cells and L929 cells, respectively. As shown in Fig. 4a and b, 4T1 cells were significantly destroyed by MnCO_3_ and PEI-MnCO_3_ NCs (p < 0.05) compared to L929 cells under the same condition, which illustrated the TME-responsive characters and selective toxicity of PEI-MnCO_3_ NCs on cancer cells. Moreover, we investigated the cancer-killing effects of MnCO_3_-based NCs, i.e. PEI-MnCO_3_, Rf, MRf, and MRp in dark or under LED light. As expected, MRp + LED light exhibited the highest toxicity to 4T1 cells, suggesting CDT, PDT and pDNA comprised an enhanced tumor therapeutic efficacy (Fig. 4c). Furthermore, the live/dead staining was employed to investigate the cell status with different treatments. As compared to normal cells, 4T1 cells suffered from different levels of damage in the MnCO_3_-related groups. Specifically, all the cancer cells in MRf + light group became red (death) and round, the results further confirmed their excellent tumor-killing effect (Fig. 4d).

### Intracellular distribution and characters in 4T1 cells

**Fig. 5:**
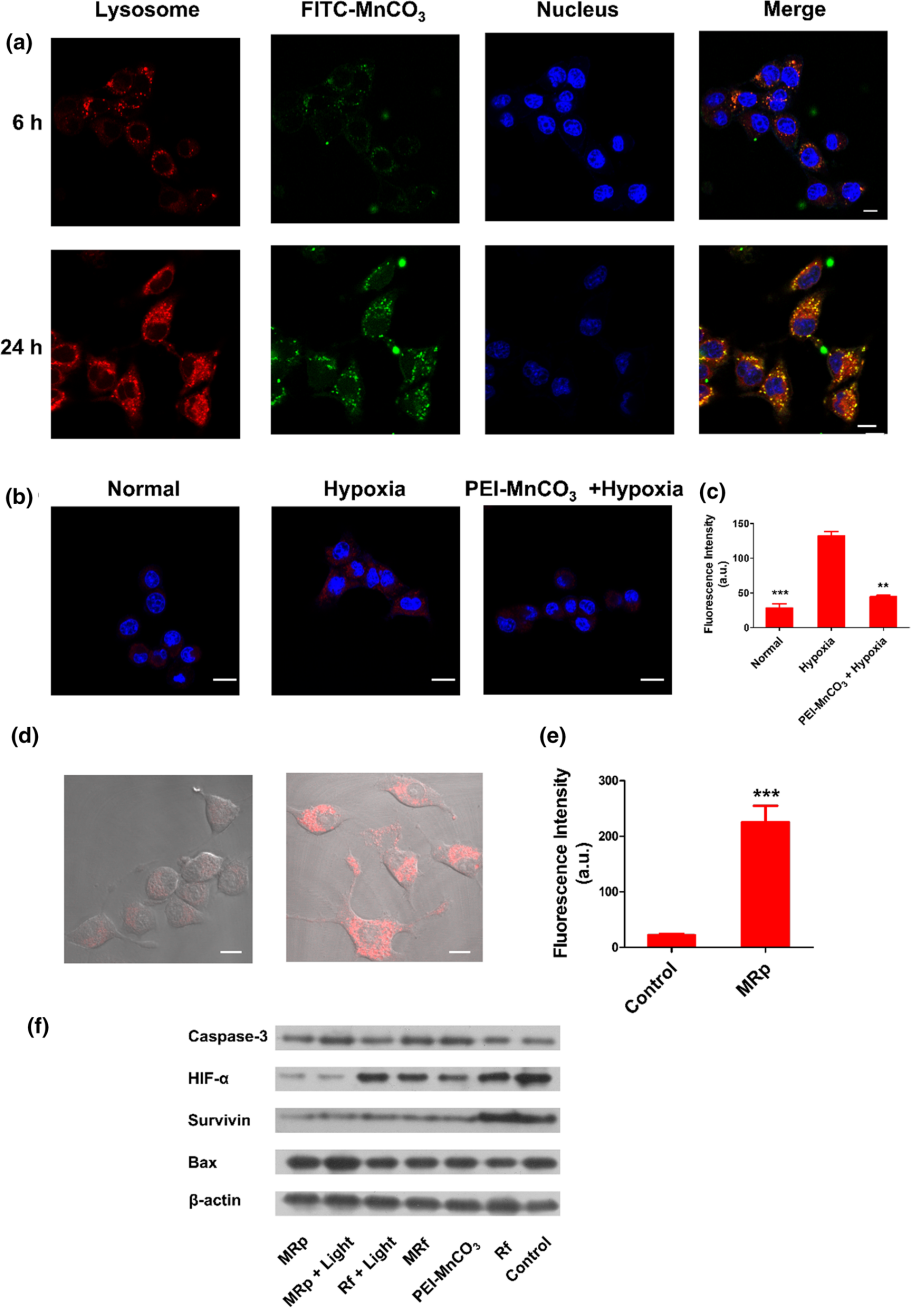
Behaviors of MnCO_3_ in 4T1 cells. **a** Distribution of FITC-MnCO_3_ NCs in 4T1 cells, lysosome (red), MnCO_3_ (green), Hochest 33342 (blue), the scale bar in the above images is 10 μm. **b** Fluorescence images of 4T1 cells treated with PEI-MnCO_3_ NCs for relief of hypoxia. **c** Fluorescence intensity of hypoxia-probe (Ex/Em = 488 nm/580 nm) in different groups. **d** Fluorescence images of 4T1 cells after transfected with MRp (the p-DNA was labeled with firefly luciferase (FLUC)). Left: control cells; right: MRp transfected cells. **e** Fluorescence intensity of FLUC probe in 4T1 cells (Em = 540 nm). Data are represented as mean ± SD; n = 4; Statistical significance was analyzed by the two-tailed Student’s *t*-test. *p < 0.05, **p < 0.01. **f** Western bolt results of the target protein expression after different treatments in 4T1 cells

Because of the high killing efficiency of MnCO_3_-based NCs, we next investigated the behaviors of NCs in 4T1 cells. As shown in Fig. 5a, the FITC-MnCO_3_ NCs was distributed in the cytoplasm, some of them around the lysosomes, with an overlap coefficient of 50% with the lysosome at the first 6 h; With the increase of time, the FITC-MnCO_3_ NCs fluorescence area covers 81% of lysosomes at 24 h, illustrating a lysosome targeted effect (Additional file 1: Fig. S7). The acidic environment in lysosomes is beneficial to the degradation of MnCO_3_-based NCs, and the alkaline environment provided by MnCO_3_ NCs will destroy lysosomes, thus accelerating the death of cancer cells.

Hypoxia is an important character of solid tumors, which contribute greatly to tumor metastasis and the resistance to radio/chemotherapy [22]. Moreover, hypoxia also limited the efficiency of PDT and CDT [23]. Hypoxia-inducible factor-1*α* (HIF-1*α*) is highly active under hypoxic conditions, resulting in the changes of caspase-3 and Bax expression in tumors [24]. Here, the relief of hypoxia in PEI-MnCO_3_ was evaluated both by hypoxia probe and Western blot. As shown in Fig. 5b and c, the red fluorescence in hypoxia treatment group was significantly enhanced compared with the untreated group. In contrast, the hypoxia cells co-cultured with PEI-MnCO_3_ NCs showed weak red fluorescence, illustrating the relief of hypoxia by intracellular O_2_ generation of MnCO_3_ NCs.

Survivin is overexpressed in TNBC membranes [25], moreover, active Survivin induces the abnormal expression of several genes, including Bcl-2, Bax, and caspase-3 [26]. We evaluated the transfection properties of PEI-MnCO_3_. As shown in Fig. 5d, e, the FLUC-pDNA combined PEI-MnCO_3_ NCs exhibited stronger red fluorescence intensity than the free FLUC-pDNA group, confirming the effective transfection efficiency of PEI-MnCO_3_ NCs. Meanwhile, we examined the related protein expressions after different treatments (Fig. 5f). The results showed MnCO_3_-based NCs treatment significantly decreased the HIF-α expression, thus relieved the hypoxia status of tumors. Moreover, MRp knockdown the survivin gene in 4T1 cells. Together with the downregulation of HIF-α and survivin genes, the related pro-apoptotic proteins, caspase-3 and Bax were upregulated (Fig. 5f). These genes work together to accelerate the progress of cell apoptosis and death [27].

### Therapeutic effect in vivo

**Fig. 6:**
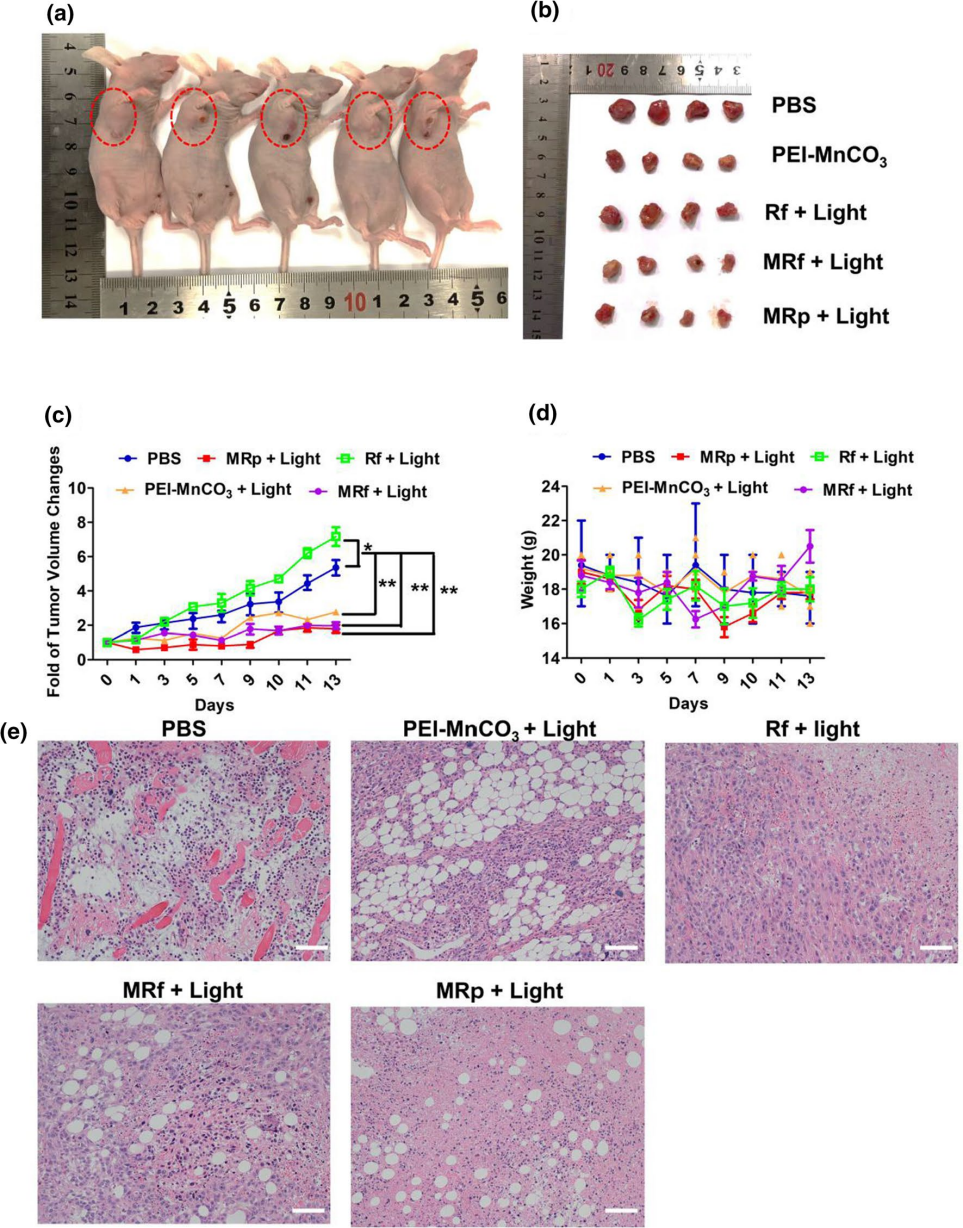
Therapeutic effect of different groups in vivo. **a** Represent images of 4T1-bearing nude mice in different groups at day 14. **b** Tumors collected from different groups of mice at day 14. **c** Tumor growth curves and (**d**) body weight changes of mice after different treatment during 14 days. **e** H&E staining of tumor sites after different treatment. Scale bar = 50 μm

The above results clearly confirm the anticancer ability of the MRp in vitro, we further evaluated their anticancer efficacy in tumor-bearing 4T1 mice. In the 4T1 tumor model (Bab/c nude mice), mice received PBS, MRp, MRf, Rf, PEI-MnCO_3_ under LED light in tumor sites when their tumor size reached to 50–70 mm^2^. Importantly, there was no thermal damage or surrounding tissue damage during the treatment process. After the treatment, the tumor size, body weight changes, and their activeness were observed every 2 days. As shown in Fig. 6a, b, the tumor volume was significantly inhibited in MRp, MRf and PEI-MnCO_3_ groups during the observed period. However, the tumor in Rf + light treatment group exhibited first restrained effect and subsequently promoted dynamic changes. This is probably because that the ROS released by Rf + LED light inhibited the tumor growth at first, then the hypoxia caused by PDT promoted tumor growth. While MnCO_3_-based groups exhibited better therapeutic effects than the control group because of the sustained TME amelioration (pH, hypoxia) and ^1^O_2_ generation. In addition, there were no notable differences in body changes among all treatment groups, and the mice in PEI-MnCO_3_ based treatment groups were active, indicating their potential biosafety. Furthermore, the hematoxylin and eosin (H&E) staining of the tumors illustrated the high complex and rich vessels in control tumor, suggesting the vigorous proliferation ability of TNBC tumors. In contrast, the tumor tissues in MRp + Light group suffered from great damage compared to other groups. Noteworthy, there were lots of bubbles-like destruction in tumor sites after being treated with MnCO_3_ based nanomaterials, suggesting the sustained CO_2_ and O_2_ generation could result in serious tumor destruction.

**Fig. 7:**
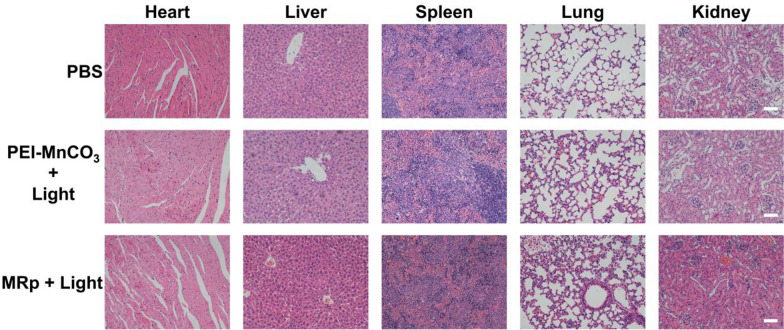
H&E staining of the main organs in PBS, PEI-MnCO_3_ + Light, and MRp + Light group. Scale bar = 50 μm

H&E staining of main organs (heart, liver, spleen, lung, and kidney) was performed after different treatments. As shown in Fig. 7, the myocardial cells and the glomeruli were intact and clear in the treatment groups. The glomerulus the hepatocytes and splenocytes were normal, and no damage or inflammatory was observed in the examined organs relative to the control group.

## Conclusions

In summary, the mesoporous PEI-MnCO_3_ NCs serve as drug loading (Rf) and transfection system (pDNA) for efficient TNBC therapy have been established because of their porous structure and positive zeta potential. Importantly, the PEI-MnCO_3_ NCs possessed TME-responsive characters, O_2_ generation ability and Mn^2+^ mediated CDT. Significantly, the ROS production ability could be amplified and the suvivin gene was silenced by MRp, which efficiently inhibited TNBC growth both in vitro and in vivo. Interestingly, the bubble (O_2_ and CO_2_) produced in the therapeutic process also destroyed the tumor tissue severely, which may provide a new idea for tumor therapy.

## Supplementary Information

**Additional file 1: Fig. S1.** The mean hydrodynamic diameter of MnCO_3_ and MRp measured by DLS. **Fig. S2.** Degeneration of PEI-MnCO_3_ under simulated TME solution. (a) Schematic illustration of PEI-MnCO_3_ degradation in TME; (b) TEM images of PEI-MnCO_3_ NCs under TME at different time intervals. **Fig. S3.** O_2_ production in different concentrations of commercial MnCO_3_, O_2_ contents was detected using a portable dissolved oxygen meter (HANNA HI 2400). **Fig. S4.** CO_2_ generation ability of PEI-MnCO_3_ NCs in simulated TME (2 mM H_2_O_2,_ pH = 5.5) solution. **Fig. S5.** XRD spectra of PEI-MnCO_3_ degradation in simulated TME solution. **Fig. S6.** XPS spectra of PEI-MnCO_3_ NCs after incubated in simulated TME (2 mM H_2_O_2,_ pH = 5.5) solution for 2 h. (a) Full XPS spectrum of PEI-MnCO_3_ NCs. XPS spectra of (b) Mn and (c) O. **Fig. S7.** Pearson’s coefficient of PEI-MnCO_3_ NCs overlap lysosome (From Fig. 5a). Data are represented as mean ± SD; n = 4; Statistical significance was analyzed by the two-tailed Student’s *t*-test. *p < 0.05, **p < 0.01.

## Data Availability

All data generated or analyzed during this study are included in this published article and the Additional Information.
