# Supplementary material for: Biodegradable mesoporous manganese carbonate nanocomposites for LED light-driven cancer therapy via enhancing photodynamic therapy and attenuating survivin expression
Source: J Nanobiotechnology. 2021 Oct 9;19:310. doi: 10.1186/s12951-021-01057-2 (PMC8502371; doi:10.1186/s12951-021-01057-2)
Supplement: Supplementary file 1 — Additional file 1: Fig. S1. The mean hydrodynamic diameter of MnCO3 and MRp measured by DLS. Fig. S2. Degeneration of PEI-MnCO3 under simulated TME solution. (a) Schematic illustration of PEI-MnCO3 degradation in TME; (b) TEM images of PEI-MnCO3 NCs under TME at different time intervals. Fig. S3. O2 production in different concentrations of commercial MnCO3, O2 contents was detected using a portable dissolved oxygen meter (HANNA HI 2400). Fig. S4. CO2 generation ability of PEI-MnCO3 NCs in simulated TME (2 mM H2O2, pH = 5.5) solution. Fig. S5. XRD spectra of PEI-MnCO3 degradation in simulated TME solution. Fig. S6. XPS spectra of PEI-MnCO3 NCs after incubated in simulated TME (2 mM H2O2, pH = 5.5) solution for 2 h. (a) Full XPS spectrum of PEI-MnCO3 NCs. XPS spectra of (b) Mn and (c) O. Fig. S7. Pearson’s coefficient of PEI-MnCO3 NCs overlap lysosome (From Fig. 5a). Data are represented as mean ± SD; n = 4; Statistical significance was analyzed by the two-tailed Student’s t-test. *p < 0.05, **p < 0.01. [file 12951_2021_1057_MOESM1_ESM.docx]

Supporting Information

**Biodegradable Mesoporous Manganese Carbonate Nanocomposites for LED Light-Driven Cancer Therapy *via* Enhancing Photodynamic Therapy and Attenuating Survivin Expression**

Lihua Li^1^, Lingling Chen^2^, Ling Huang^1^, Xiangling Ye^3^, Zefeng Lin^2^, Xiaoming Wei^1^, Xianfeng Yang^1^*, and Zhongmin Yang^1^*

Fig. S1 The mean hydrodynamic diameter of MnCO_3_ and MRp measured by DLS.

Fig. S2. Degeneration of PEI-MnCO_3_ under simulated TME solution. (a) Schematic illustration of PEI-MnCO_3_ degradation in TME; (b) TEM images of PEI-MnCO_3_ NCs under TME at different time intervals.

Fig. S3. O_2_ production in different concentrations of commercial MnCO_3_, O_2_ contents was detected using a portable dissolved oxygen meter (HANNA HI 2400).

Fig. S4. CO_2_ generation ability of PEI-MnCO_3_ NCs in simulated TME (2 mM H_2_O_2,_ pH=5.5) solution.

Fig. S5. XRD spectra of PEI-MnCO_3_ degradation in simulated TME solution.

Fig. S6. XPS spectra of PEI-MnCO_3_ NCs after incubated in simulated TME (2 mM H_2_O_2,_ pH=5.5) solution for 2 h. (a) Full XPS spectrum of PEI-MnCO_3_ NCs. XPS spectra of (b) Mn and (c) O.

Fig. S7. Pearson’s coefficient of PEI-MnCO_3_ NCs overlap lysosome (From Fig. 5a). Data are represented as mean ± SD; n=4; Statistical significance was analyzed by the two-tailed Student’s *t*-test. *p<0.05, **p<0.01.
